# Bioactivities of *Centaurium erythraea* (Gentianaceae) Decoctions: Antioxidant Activity, Enzyme Inhibition and Docking Studies

**DOI:** 10.3390/molecules24203795

**Published:** 2019-10-22

**Authors:** Laura Guedes, Pedro B. P. S. Reis, Miguel Machuqueiro, Asma Ressaissi, Rita Pacheco, Maria Luísa Serralheiro

**Affiliations:** 1 Faculty of Sciences, BioISI—Biosystems & Integrative Sciences Institute, University of Lisbon, 1649-004 Lisboa, Portugal; lrguedes@fc.ul.pt (L.G.); aressaissi@fc.ul.pt (A.R.); rpacheco@deq.isel.ipl.pt (R.P.); 2 Faculty of Sciences, CQB—Centre of Chemistry and Biochemistry, University of Lisbon, 1649-004 Lisboa, Portugal; pdreis@ciencias.ulisboa.pt (P.B.P.S.R.); machuque@ciencias.ulisboa.pt (M.M.); 3 Área Departamental de Engenharia Química, Instituto Superior de Engenharia de Lisboa, Av. Conselheiro Emídio Navarro, 1959-007 Lisboa, Portugal; 4 Faculty of Sciences, Departamento de Química e Bioquímica, University of Lisbon, Campo Grande, 1749-016 Lisboa, Portugal

**Keywords:** *Centaurium erythraea*, acetylcholinesterase, HMG-CoA reductase, gentiopicroside, xanthones, docking studies

## Abstract

*Centaurium erythraea* is recommended for the treatment of gastrointestinal disorders and to reduce hypercholesterolemia in ethno-medicinal practice. To perform a top-down study that could give some insight into the molecular basis of these bioactivities, decoctions from *C. erythraea* leaves were prepared and the compounds were identified by liquid chromatography-high resolution tandem mass spectrometry (LC–MS/MS). Secoiridoids glycosides, like gentiopicroside and sweroside, and several xanthones, such as di-hydroxy-dimethoxyxanthone, were identified. Following some of the bioactivities previously ascribed to *C. erythraea*, we have studied its antioxidant capacity and the ability to inhibit acetylcholinesterase (AChE) and 3-hydroxy-3-methylglutaryl coenzyme A reductase (HMGR). Significant antioxidant activities were observed, following three assays: free radical 2,2-diphenyl-1-picrylhydrazyl (DPPH) reduction; lipoperoxidation; and NO radical scavenging capacity. The AChE and HMGR inhibitory activities for the decoction were also measured (56% at 500 μg/mL and 48% at 10 μg/mL, respectively). Molecular docking studies indicated that xanthones are better AChE inhibitors than gentiopicroside, while this compound exhibits a better shape complementarity with the HMGR active site than xanthones. To the extent of our knowledge, this is the first report on AChE and HMGR activities by *C. erythraea* decoctions, in a top-down analysis, complemented with *in silico* molecular docking, which aims to understand, at the molecular level, some of the biological effects ascribed to infusions from this plant.

## 1. Introduction

*Centaurium erythraea* (*C. erythraea*) is a plant belonging to the Gentianaceae family, known as common centaury, growing in almost all Europe, North Africa and Southwest Asia [1]. It is a medicinal plant with several applications, among which gastrointestinal motility [1], gastric damage [2] as well as reduction of total blood cholesterol [3] can be mentioned. The European Medicine Agency (EMA) has a report on its applications [4] and in China a secoiridoid present in this plant was approved as a new drug to treat acute jaundice and chronic active hepatitis [5].

The gastro-protective effect observed with *C. erythraea* aqueous-ethanol extracts could, in part, be explained by its antioxidant activity [2,6]. Radical species can be formed inside cells due to several oxidation processes. These reactive oxygen and nitrogen species induce modifications in DNA, protein, and lipid molecules, which can lead to severe cell damage [7]. Cells have several repairing mechanisms to deal with these damaged molecules and many antioxidant compounds that avoid radical formation or promptly neutralize their effect [8,9]. Several diseases are related to oxidative processes in cells [10,11]. 

Aerial parts of *C. erythraea* have shown antimicrobial [12], gastro-protective [2] properties, and gentiopicroside from C.erythraea has shown anti-inflammatory effects [13], without exhibiting toxicity towards laboratory animals (rodents) [14]. Extracts from this plant are rich in secoiridoid glycosides, namely gentiopicroside, sweroside, swertiamarin, and in xanthone derivatives [15,16]. Several biological activities have been attributed to gentiopicroside, namely inhibition of the spontaneous contraction of the ileum smooth muscle [17], including analgesic, anti-inflammatory and anti-acetylcholinesterase activities [18,19]. This iridoid glycoside offers hepatoprotection against compounds that produce liver failure [20] and can also protect DNA from damage [21]. Gentiopicroside was able to increase gastrointestinal motility by modifying the expression of several hormones related with intestinal propelling [22] as well as reducing the total cholesterol level in laboratory animals [23]. Xantones, which are also present in *C. erythraea*, have shown several biological effects, including antitumor and anti-inflammatory activities [24,25]. Xanthone derivatives can inhibit acetylcholinesterase (AChE) and block the acetylcholinesterase-induced β-amyloid aggregation [26]. These compounds are also known for their antiproliferative effect and their ability to inhibit several molecular targets in the tumor cells, including kinases, cyclooxygenases and DNA polymerases [8].

The plant extracts effects mentioned, like gastro-intestinal propelling and blood cholesterol reduction, in addition to its association with Alzheimer’s Disease, have guided us to evaluate the activities of AChE and 3-hydroxy-3-methyl-glutaryl-coenzyme A reductase (HMGR). AChE (EC 3.1.1.7) is an enzyme located in the synaptic clefts and neuromuscular junctions [26] that catalyzes the hydrolysis of the neurotransmitter acetylcholine, which is essential for neuronal connectivity and junction contraction mechanism [27]. AChE has also been shown to influence gastrointestinal and colon motility [28] and its reversible inhibition can help treat gastrointestinal (GI) disorders [19,20,21,22,23,24,25,26,27,28,29,30,31] and Alzheimer’s disease [32]. HMGR (EC 1.1.1.34) enzyme is an important regulator of the cholesterol biosynthesis pathway [33] and its inhibition by statins has been used therapeutically to reduce total blood cholesterol levels [34].

The aim of this study is to identify the key compounds present in *C. erythraea* decoction, a common way of consuming herbal beverages, and to test for its antioxidant effects, and for AChE and HMGR inhibitory activities, which can help explain the decoctions impact in ethno-medicine [1]. In this top-down approach, we will also perform computational molecular docking calculations to help rationalize the molecular details of the mechanisms of action and pinpoint the individual contributions of each compounds. In overall, this study contributes to the scientific knowledge on the mechanisms of action of *C. erythraea* decoctions, an herb described by the Committee on Herbal Medicinal Products (HMPC, from EMA).

## 2. Results and Discussion

### 2.1. Decoction Phenolic Compounds

**Figure 1:**
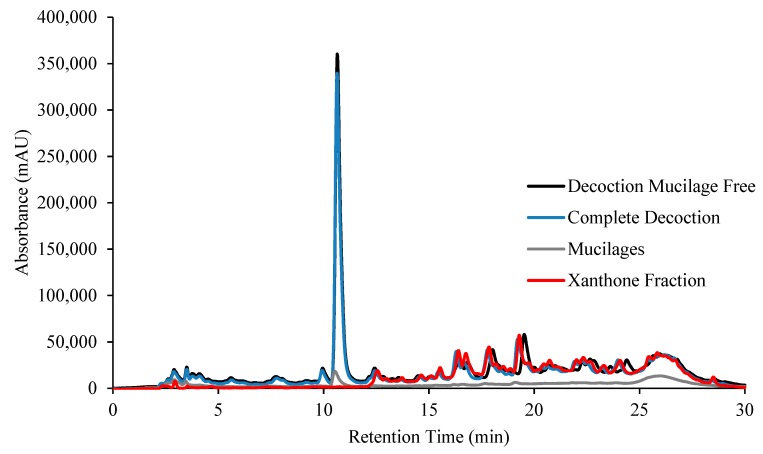
Reversed-phase high-performance liquid chromatography with diode array detector (RP-HPLC–DAD) chromatogram of decoction of *C. erythraea* and its isolated fractions.

Decoctions of *C. erythraea* were prepared and analyzed by reversed-phase high-performance liquid chromatography with diode array detector (RP-HPLC–DAD), Figure 1. Since water can extract sugar polymers, ethanol precipitation of these compounds (mucilages’ precipitation) was carried out and the supernatant decoction mucilage free (DM_f_) was analyzed by RP-HPLC–DAD, Figure 1. The amount of precipitated material was very small (only 5.5%), meaning that *C. erythraea* has a very low amount of sugar polymers. The pellet did not have phenolic compounds since no peaks were seen in the chromatogram, Figure 1. In this chromatogram one major peak is detected around 11 min and several compounds appear after this retention time. It can be seen that the decoction without mucilages overlap with the complete extract and the pellet has no detectable compounds.

**Figure 2:**
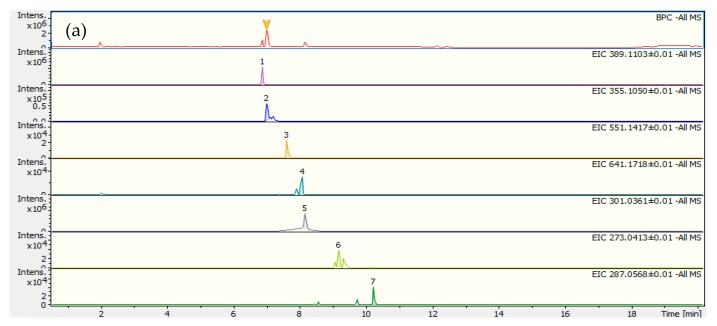

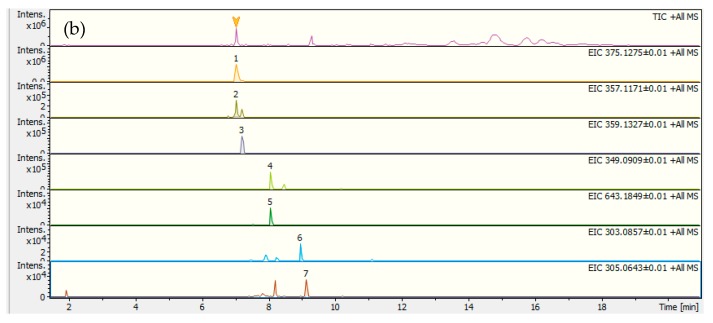
Total ionic chromatogram and ionic chromatograms extracted from ions identified in *C. erythraea* complete decoction: (**a**) liquid chromatography–electrospray ionization tandem mass spectrometry (LC–ESI (−) MS/MS) and (**b**) LC–ESI (+) MS/MS. Peaks 1-7 are identified in Table 1.

In order to identify the compounds LC–MS/MS was carried out. LC–MS in the negative and positive mode (Figure 2a,b, respectively) and LC–MS/MS allowed the identification of the compounds indicated in Table 1, either by using information from the literature or by taking into account the exact mass. The first chromatogram in Figure 2a,b, is the BPC chromatogram with all the compounds present in the complete extract. This chromatogram is identical to that obtained by RP-HPLC–DAD, that is a major compound followed by lots of several compounds with lower intensity. The identification of the compounds indicated the presence of five secoiridoids, six xanthones and one flavonoid (quercetin). The major compound is gentiopicroside, a secoiridoid structure compound characteristic of the Gentianaceae family to which *C. erythraea* belongs [35]. The compounds with retention time between 8 and 30 min were identified as xanthones derivatives and quercetin, Table 1. Before this retention time most of the compounds had the same structure type of secoiridoids. Due to the similarity between the chromatograms and to the use of the standard compound gentiopicroside, the peak with the highest intensity in RP-HPLC–DAD (Figure 1) contains the secoiridoid derivatives. The small peaks at higher retention time belong to xanthone type compounds, according to the mass identification results. 

The separation between xantones and iridoids was relatively easy due to their structural differences. A fraction containing mainly xanthones (XF) was obtained by repeatedly injecting the DM_f_ in the HPLC–DAD and collecting only the compounds coming out the column between 11.5 and 30 min. This fraction was injected again in the chromatographic system to confirm that the compounds belonged to the xanthone fraction in Figure 1.

Since several biological activities have been attributed to either gentiopicroside or xanthone derivatives, we have complemented the analysis of the *C. erythraea* complete decoctions with studies of DM_f_ and XF.

### 2.2. Total Phenols Quantfication

Total phenols were quantified in all samples, Table 2, and the amounts detected by the Folin–Ciocalteu method were quite low. This was expected since this method quantifies the phenol hydroxyl groups, which are absent in some of the identified compounds from the HPLC–DAD and LC–MS/MS results, such as gentiopicroside, swertiamarin or sweroside. The aqueous extract (D) has the highest amount of phenolic compounds mesasured as Gallic Acid Equivalents (GAE) (22.37 ± 0.36 μg GAE/mg extract), followed by DM_f_ (18.82 ± 0.25 μg GAE/mg extract) and finally XF (16.31 ± 0.25 μg GAE/mg extract). These values were statistically different at *p* ≤ 0.05.

### 2.3. Antioxidant Activity

In recent years, there has been a great deal of attention towards natural antioxidants that can be used in our diet and help the organism counteract the oxidative stress. To evaluate the biological activities of the compounds present in the decoctions, we used both XF and the standard gentiopicroside to represent the most abundant compound of the chromatogram. The most widely used method to measure antioxidant activities is the 2,2-diphenyl-1-picrylhydrazyl (DPPH) scavenging radical test [36], but due to some reported drawbacks of this method [37] and to the complexity of antioxidant mechanisms inside cells, we complemented our study with other fast and inexpensive in vitro methodologies, which model the in vivo antioxidant processes. In these assays, the antioxidant potential of *C. erythraea* was evaluated by its ability to reduce the DPPH radical, to inhibit lipid peroxidation and to capture nitric oxide radicals avoiding its reaction with biological molecules.

#### 2.3.1. DPPH (2,2-Diphenyl-1-Picrylhydrazyl) Radical Scavenging Activity 

As shown in Table 2, using the same concentration (50 μg/mL) D is the one with the highest percentage of free radical capture (approximately 25%) while the DM_f_ has a radical reduction percentage of ~22%. These results suggest that during the mucilage precipitation process some antioxidant compounds are retained in the mucilages or that some constitutive compounds of mucilages have the ability to reduce the DPPH radical. XF has a good antioxidant activity compared to the complete decoction; 68% of the DPPH scavenging capacity in the complete decoction corresponds to the xanthone fraction or 75% relative to the DM_f_. This suggests a low antioxidant activity of secoiridoids detected in this decoction. As expected, the antioxidant activities measured correlate well with the amount of total phenols quantified in the different samples (r^2^ = 0.9677). Based on the results obtained by LC–MS/MS, we propose that most xanthones have hydroxyl groups in their structure that can easily provide electrons and quench the DPPH radical. 

The gentiopicroside standard showed a low antioxidant activity (~1%), while for commercial butylated hydroxytoluene (BHT), rutin and quercetin the EC_50_ values are 9.89 μg/mL, 6.27 μg/mL, and 3.23 μg/mL, respectively. The gentiopicroside result was expected since this compound has no radical stabilizing group, hence it is unable to donate electrons to the radical form of DDPH.

It is noteworthy that studies carried out with *C. erythraea* secoiridoides revealed that neither swertiamarin nor sweroside, identified by LC–MS/MS in this work, have antioxidant activity [12]. Therefore, these data combined with the practically non-existent activity of gentiopicroside may explain the low antioxidant activity of this extract when compared to the commercial standards mentioned above. Furthermore, our antioxidant activity results are in good agreement with other plant extract having similar compounds [38,39].

#### 2.3.2. Lipoperoxidation Inhibition 

The antioxidant activity measured as TBARs formation indicates a capacity to counteract lipid oxidation. As presented in Table 2, for the final concentration of 400 μg/mL D has an inhibition of approximately 32%, which is lower than that of BHT, the commercial standard (49.09 μg/mL). This activity is lower than the one obtained with plant-water extracts containing polyphenols [40]. In *C. erythraea* extracts, secoiridoids and xanthones are the main components and although xanthones with free hydroxyl groups have the capacity to avoid lipid peroxidation [41], most of those detected in our study have several hydroxyl groups methylated or functionalized with sugar moieties. These modifications reduce significantly the number of free hydroxyl groups available, which are indeed the source for the antioxidant activity detected in the present work. 

#### 2.3.3. Nitric Oxide Radical Scavenging 

NO^•^ is a free radical species naturally generated in the cell with important physiological functions. However, it becomes a hazardous compound when it reacts with superoxide radical, forming the peroxynitrite anion (ONOO^−^), a powerful oxidant [42]. The NO^•^ radical scavenging activity is a measure of peroxynitrite elimination, hence, of avoiding oxidative stress in the cell. In this method NO^•^ is formed from sodium nitroprusside and it reacts with molecular oxygen to produce nitrate and nitrite, the latter being quantified using Griess reagent [43]. Using this method to model NO^•^ synthesis in the cell, we can measure the radical scavenging ability of the decoction, Table 2. In this study the aqueous extract (D) has a lower inhibitory capacity (EC_50_ = 775 μg/mL) than rutin (EC_50_ = 314 μg/mL), a potent antioxidant known for its NO^•^ scavenging ability. Nevertheless, decoctions containing a mixture of flavonoid derivatives [43] have shown a higher activity, suggesting that neither secoiridoids nor xanthones have a significant capacity for capturing the NO radical.

### 2.4. Enzyme Inhibition 

#### 2.4.1. Acetylcholinesterase (AChE) Inhibitory Activities and Docking Studies 

*C. erythraea* is used as decoctions or infusions to improve the food digestive process [22]. One way of improving this digestive process is increasing the intestinal transit, which can be accomplished by inhibiting the enzyme acetylcholinesterase (AChE) in the neuromuscular junctions. This mechanism has been shown to accelerate the gastrointestinal motility in post-operated human patients and animals [28,30]. The AChE inhibition assays were performed using D, DM_f_, XF and gentiopicroside standard. No correlation was observed between the amount of total phenols present in the fractions and the enzyme inhibitory activity (r^2^ = 0.0256), which was expected since there are many factors that influence the inhibition constant of AChE and most of them are not related with the number of phenol groups in the compound. Furthermore, not all compounds are able to fit inside the enzyme active site in the most effective way.

For the final concentration of 500 μg/mL, the sample that showed a higher inhibitory activity was XF (~60%), followed by D (~56%) and by the DM_f_ (~47%). Previous studies with 1,5,8-trihydroxy-3-methoxyxanthone (isolated from *Gentiana campestris*, also belonging to the Gentianaceae family) showed enzyme inhibition values very similar to the commercial standard galantamine [44], highlighting the role these compounds can play in the treatment of digestive problems and even Alzheimer´s Disease. Due to the low activity of D containing the major component gentiopicroside relatively to the XF, the standard compound was also added in this study and its AChE inhibitory activity determined, Table 2. The AChE inhibition using the pure compound, at 500 μL/mL, was very low (6.5%), confirming its poor contribution compared with the standard galantamine (0.14 μg/mL) or donepezil (2 ng/mL) [45].

**Figure 3:**
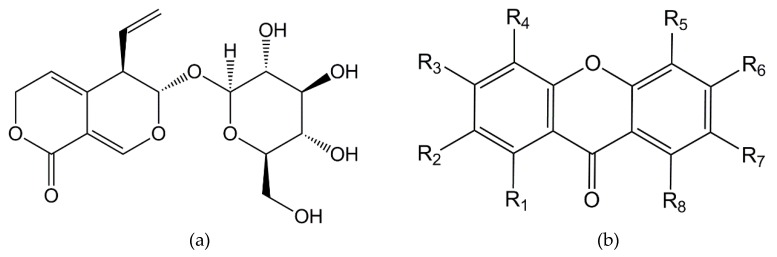
Schematic structures: (**a**) gentiopicroside; (**b**) xanthone (template). The different substitutions in the R1–R8 positions, leading to the xanthone molecules used in the docking protocol, are described in Table 3.

The clear differences in inhibition capacities of AChE by gentiopicroside and xanthone led us to investigate the compound/enzyme interactions at the molecular level. We performed computational docking calculations of the poor inhibitor gentiopicroside and several xanthone derivatives (Figure 3) that comply with the molecular weight reported by the LC–MS/MS assay (Table 1). We divided the tested xanthones into three classes: xanthones previously described in the literature (Table 3 xan01-11), rationally designed xanthone compounds (Table 3 xan12-15) and xanthone derivatives from the most potent inhibitors of the two previous groups (Table 3 xan08.1-15.1). Xanthones identified in the present work are indicated in Table 3 by the corresponding peak number in Table 1. In this study several hypotheses were proposed for the localization of OH and OMe groups according to different identifications reported in the literature. This fact attributes several compounds for the same peak identified by LC–MS/MS. 

**Figure 4:**
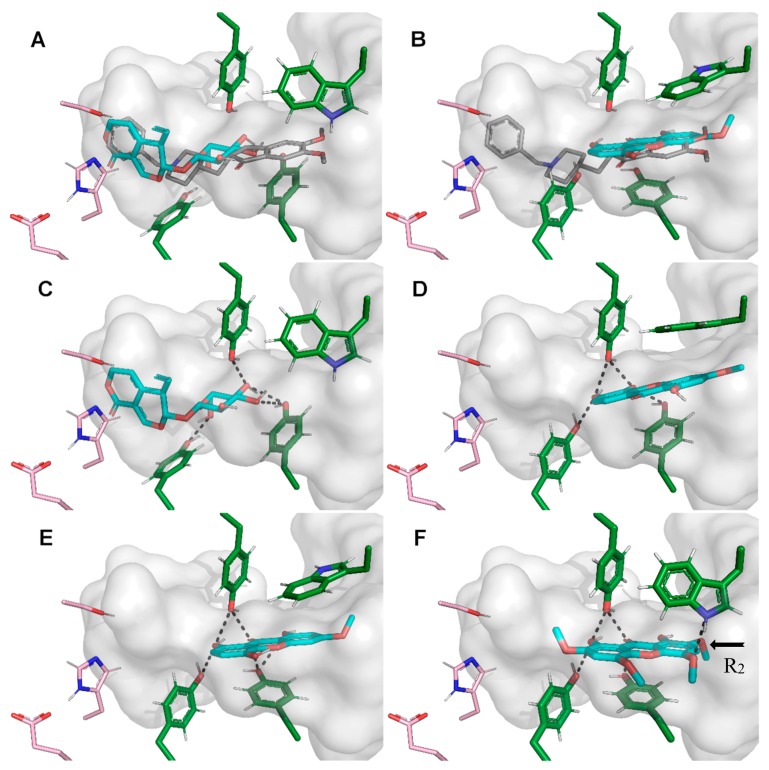
The lowest energy solutions from the docking calculations of gentiopicroside and the most active xanthones (cyan) in human AChE (green) are presented. Gentiopicroside (**A**) and xanthone 04 (**B**) are presented along with the X-ray structure of donepezil (PDB ID: 4EY7; [47]; dark grey) for a direct comparison. The interaction maps (black dashed lines) of gentiopicroside (**C**) and xanthones (**D**—xan03, **E**—xan04, **F**—xan09) in their best poses are also shown. The most active xanthone (xan09.1) differs from xan09 by removal of a methoxy group in the position R_2_ (highlighted with a black arrow—**F**). The catalytic residues of AChE are indicated with pink carbons. The active site pocket was calculated with hollow software [48] and represented as a gray surface.

The docking calculations that were performed show that these two compound families bind to distinct pocket regions. Gentiopicroside binds in a deeper position, near the catalytic triad of AChE, while xanthones bind at the access tunnel gorge (Figure 4A,B). The two binding positions are complementary and together resemble donepezil binding mode. Like galantamine, donepezil is a very potent AChE inhibitor and we expected that neither gentiopicroside nor any xanthone can be as strong inhibitors, because these only exhibit a fraction of the shape-complementarity and the interactions donepezil establishes in the pocket of AChE. According to our simulations, gentiopicroside is a modest AChE inhibitor (calculated K_i_ = 62.3 μM) as a consequence of not interacting strongly with the enzyme. We observe only a few individual hydrogen bonds between the glycoside group and the tyrosine side chains, while the rest of the gentiopicroside molecule binds near the catalytic triad without establishing noticeable direct interactions (Figure 4C). 

The preferred binding mode of most xanthones is at the access tunnel of the active site where the compounds seem to maximize the number of interactions and present a strong shape complementarity. The low polarity of this class of molecules leads to an increased binding affinity obtained from desolvation. From all evaluated xanthones, xan08, xan09, xan12 and xan12.1 (Figure 4D–F) presented significantly strong inhibition constants (Table 3). Unlike gentiopicroside, xanthones are able to establish a higher number of hydrogen bonds, including a network of shared bonds with the previously mentioned tyrosine side chains. The size of this hydrogen network may explain the differences between the strong and the not so strong inhibitors (Table 3). Position R_1_ seems to be the most important substitution for the formation of referred hydrogen network, which becomes particularly strong with a hydroxyl group, behaving both as a hydrogen bond acceptor and donor. Position R_5_ is also important since it interacts with the protein backbone, either as a donor (with carbonyl) or acceptor (with amide). Compound xan12 is able to establish an extra hydrogen bond with Trp286 (Figure 4F) which was retained in the binding mode of xan12.1, a derivative obtained by removing the methoxy group in position R_2_ (the arrow in Figure 4F), the strongest inhibitor found in our computational study. 

The docking studies that were performed showed that unlike gentiopicroside, xanthones which are also present in *C. erythraea* decoction, can be strong AChE inhibitors and their inhibition power depends on their R_x_ group substitutions. Furthermore, in our work xan09 was identified as a good inhibitor, which is in good agreement with the experimentally measured IC_50_ value of 18.5 μM [49]. A series of rationally designed xanthones were created based on the best inhibitors found in this study and xan12.1 was identified as the strongest inhibitor we observed. However, stronger xanthone-based inhibitors with this template may still be undiscovered since we only grasped a fraction of all possible combinations of chemical groups and substitution positions. Therefore, our molecular docking protocol is particularly useful estimating the inhibition constant value range of a large number of xanthones, especially considering no specific xanthone identification was performed in the extracted fraction of *C. erythraea* decoction. Furthermore, our docking studies can help to foresee the location or position of the substituents in the xanthones identified in the extract as a tentative identification, according to the high activities determined.

#### 2.4.2. HMGR (3-hydroxy-3-methylglutaryl coenzyme A reductase) Inhibitory Activities and Docking Studies 

*C. erythraea* has also been reported to reduce high levels of blood cholesterol [4]. In the clinic, high levels of blood cholesterol are treated with statins, known to be cholesterol biosynthesis inhibitors. They act by inhibiting the activity of the regulator enzyme in the cholesterol biosynthetic pathway, 3-hydroxy-3-methyl-glutaryl-coenzyme A reductase (HMGR). 

The inhibition of this enzyme was tested with D (48%) and DM_f_ (27%), for the same concentration of 10 μg/mL. These results suggest that the inhibitory potential of *C. erythraea* is mainly due to the monoterpenoids, which may reflect the potential of this plant in reducing cholesterol synthesis. The drug simvastatin has an IC_50_ of 0.2 μg/mL [40] which means it has a much stronger activity than the compounds present in *C. erythraea*. However, the drug is a pure compound and the decoction under study is a natural mixture of several compounds, some of which can have significant activities that are being masked in the mixture.

Studies of in silico docking report that swertiamarin, identified in this work, acts at the level of several enzymes and transcription factors involved in the metabolism of glucose and lipids, including in the inhibition of HMGR [50]. Peffley et al. [51] observed that monoterpenoids derived from the plant mevalonate pathway can control the synthesis of HMGR through a mechanism that appears to modulate the efficiency of mRNA translation. 

**Figure 5:**
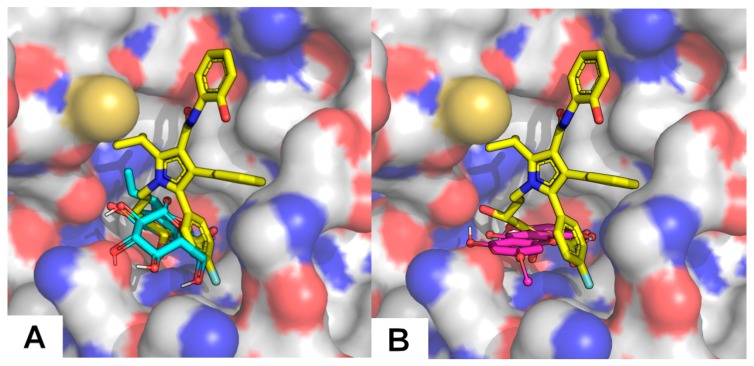
The lowest energy solutions from the docking calculations of a gentiopicroside (cyan—**A**) and xanthone 03 (pink—**B**) overlapped with the crystallographic structure of an atorvastatin derivative (yellow—1HW9); [53] in human HMGR (white carbons surface).

In order to evaluate at molecular level and assign to which type of compounds present in the extract the high activity of HMGR inhibition could be ascribed, docking studies were also performed with gentiopicroside and all xanthones previously presented. The xanthone compound family has a modest binding affinity towards HMGR, featuring computed K_i_ ranging from 54.8 to 184.8 μM (Table 4). However, gentiopicroside seems to better fill the HMGR active site and to establish more hydrogen bond interactions than xanthone compounds, resulting in a stronger inhibition (calculated K_i_ = 19.9 μM). This protein has a very shallow active site and, unlike most statins (calculated K_i_ = 3.5 nM for the example given), our compounds are only able to occupy a fraction of that site (Figure 5). Even though the tested compounds are of comparable size in comparison with stronger inhibitors such as piscidic acid, the latter being anionic is able to interact with a significant number of cationic residues (Lys735, Arg590, Lys692, Lys691), explaining its performance [45,52].

## 3. Materials and Methods 

### 3.1. Chemicals

All chemicals were analytical grade. Methanol (MeOH), iron (II)-sulfate-7-hydrate and trichloroacetic acid were obtained from Riedel-de Haën™ (Seelze, Germany) and formic acid, water and acetonitrile (ACN) from Fisher Scientific, OptimaTM (Hampton, NH, USA). Trifluoroacetic acid (TFA), potassium dihydrogen phosphate, tris(hydroxymethyl)aminomethane (Tris) and sodium chloride were bought from Merck (Darmstadt, Germany). Ethanol 96% was bought from Carlo Erba (Peypin, France), gentiopicroside from TransMIT (Hesse, Germany) and potassium chloride from Fluka (Seelze, Germany). Acetylcholinesterase (AChE), acetylcholine iodide (AChI), 5-5′-dithiobis (2-nitrobenzoic acid) (DTNB), HMG-CoA Reductase assay kit, gallic acid, 2,2-diphenyl-1-picrylhydrazyl (DPPH), butylated hydroxytoluene (BHT), quercetin, rutin and galantamine were obtained from Sigma (Barcelona, Spain). Magnesium chloride hexahydrate, thiobarbituric acid, hydrogen peroxide, sodium hydrogen phosphate and 1-butanol were bought to PanReac (Barcelona, Spain). Sodium hydroxide was obtained from JT Baker, UK and sodium nitroprusside and Griess reagent from VWR Chemicals PL.

### 3.2. Plant and Decoction Preparation

Aerial parts of *C. erythraea* Rafin. were bought in a local herbal shop (Celeiro-Dieta, Lisbon), dry, inside bags. The plant is caught at Global Positioning System (GPS) coordinates 39°30′42″ N, 8° 47′58″ O, Portugal, identified in the supplier and commercialized by a company certified for the commercialization of medicinal plants (ISO 9001/2015). A voucher specimen from the same species is kept in herbarium of the botanical garden with the number LISU253536. To obtain the aqueous extract a decoction of the plant was prepared, using 95 g of plant boiled in 950 mL of distilled water for 10 min. The decoction (D) was filtered through Whatman grade paper number 1 and lyophilized with a yield of 19.3%. Throughout the work, the extract was stored at −80 °C. 

### 3.3. Purification of Phenolic Compounds

#### 3.3.1. Mucilages’ Purification

A purification of D was carried out withdrawing mucilages by precipitating the polymers according to the method of Henriques et al. [43]. Briefly, to a 10 mg/mL solution of the lyophilized decoction was added ethanol 96% in a ratio of 1:5. The mixture was kept in the ice for 8 h and centrifuged in an Eppendorf 5415D equipment, DE, at 3500× *g* for 30 min. The supernatant free of mucilages was separated and kept for next experiments. The pellet was washed again with water and mucilages precipitated once more with ethanol as described previously. The second supernatant was mixed with the first and lyophilized. This fraction was designated as Decoction Mucilage Free (DM_f_) and it was stored at −80 °C during the experimental work.

#### 3.3.2. Xanthones Fraction Collection

The xanthones fraction (XF) was separated by HPLC–DAD by injecting repeatedly 25 μL of DM_f_ at 10 mg/mL and collecting fractions at retention time 11.5–30 min. 

### 3.4. High-Performance Liquid Chromatography with Diode Array Detector (HPLC–DAD) Analysis

The chromatographic analysis of the decoctions, the xanthones fraction collection and the enzymatic assay of 3-hydroxy-3-methyl-glutaryl-coenzyme A reductase (HMGR) were carried out in VWR-Hitachi Elite LaChrom®, equipped with a LiChroCART® RP-18, 5 μm, 250 × 4 mm, 100 Å column from Merck, autosampler L-2200, column oven L-2300 and diode array detector (DAD) L-2455. The software for data acquisition was EZChrom Elite®, Hitachi Japan. For extract analysis 1 mg/mL of each extract was used and for XF isolation 10 mg/mL of DM_f_ was used. The flow rate was 0.8 mL/min and the detection was carried out between 200 and 500 nm using DAD. For decoction analysis and XF collection the mobile phase consisted of 0.05% (v/v) of TFA in water (A) and acetonitrile (B). The elution conditions were as follows: 0 min, 92% A, 8% B; 20 min, 82% A, 18% B; 25 min, 45% A, 55% B; 28 min, 92% A, 8% B and 30 min, 92% A, 8% B. For HMGR inhibition assay the mobile phase consisted of KH_2_PO_4_ 100 mM in water (A) and MeOH (B), the gradient is described in Falé et al. [40]. The standard gentiopicroside was used to confirm the identification of the peak with higher intensity.

### 3.5. Compound Identification by Liquid Chromatography–High Resolution Tandem Mass Spectrometry (LC–MS/MS)

Samples were analyzed by liquid chromatography-high resolution tandem mass spectrometry (LC–MS-MS) using an Ultimate 3000 RSLCnano system (Thermo Fischer Scientific, Idstein, Germany) interfaced with a quadrupole time-of-flight (QqToF) Impact II mass spectrometer equipped with an electrospray source (Bruker Daltonics, Bremen, German).

Chromatography separation was carried out on a Kinetex 1.7 µm C18 100 Å, LC column 150 × 2.1 mm (Phenomenex, California, USA), at flow rate of 150 µL/min. Mobile phase consisted of 0.1% (v/v) of acid formic in water (A) and 0.1% (v/v) of acid formic in acetonitrile (B), the elution conditions were described in André et al. [54]. The column and the sampler were maintained at 35 °C and 10 °C, respectively.

The high-resolution mass spectra were acquired in the electrospray ionization (ESI) positive/negative modes. The optimized parameters were set as follows: ion spray voltage, +4.5/−2.5 kV; end plate offset, 500 V, nebulizer gas (N_2_), 2.8 bars; dry gas (N_2_), 8 L/min; dry heater, 200 °C. Internal calibration was performed on the high-precision calibration mode (HPC) with a solution of sodium formate 10 mM introduced to the ion source via a 20 µL loop at the beginning of each analysis using a six-port valve. Acquisition was performed in full scan mode in the *m/z* 50–1300 range, and in a data-depending MS/MS mode, with an acquisition of 3 Hz using a dynamic method with a fixed cycle time of 3 s. Dynamic exclusion duration was 0.4 min. The acquired data were processed by DataAnalysis 4.1 software (Bruker Daltoniks).

### 3.6. Total Phenol Content Quantification

Total phenol content was measured spectrophotometrically, at 760 nm, using Folin–Ciocalteu reagent as described in Henriques et al. [43], using gallic acid as a standard (10–500 μg/mL). The samples analyzed were D, DM_f_ and XF. The concentration of total phenols was determined as μg of gallic acid equivalents per mg of dry extract as the mean of three replicates.

### 3.7. Antioxidant Activity Determination

#### 3.7.1. DPPH Radical Scavenging Activity

The radical scavenging activity of *C. erythraea* was evaluated by using DPPH method described by Falé et al. [55]. In short, 10 μL of the sample solution was added at a concentration of 5 mg/mL to 1 mL of the DPPH solution (0.002% in MeOH), the solutions were incubated at room temperature for 30 min and then the absorbance of the different samples was measured on the microplate reader at 517 nm against a blank. BHT, rutin and quercetin were used as standards and for these compounds the concentration value to accomplish an extinction of absorption of 50% (EC_50_) was determined. All assays were performed in triplicate and are presented as mean and standard deviation associated to the measurements. In order to calculate the percentage of antioxidant activity, the following expression was used:

AA (%) = 100 × (A_test_ − (A_sample_/A_test_)) (1)

In which AA (%) corresponds to the percentage of antioxidant activity, A_sample_ refers to the absorbance of each sample and A_test_ to the absorbance of the control solution.

#### 3.7.2. Lipoperoxidation Inhibition

Inhibition of lipid oxidation was evaluated by the thiobarbituric acid reactive substances (TBARS) method described by Tokur et al. [56] with minor optimizations, being the values presented as the mean of three independent tests and the standard deviation associated to the measurements. In this assay, the commercial standard used was BHT and its EC_50_ determined. The percentage inhibition of lipid peroxidation was calculated according to Equation (1).

#### 3.7.3. Nitric Oxide Radical Scavenging Activity 

Nitric oxide radical scavenging assay was performed in triplicate according to the modified method described by Sakat et al. [57] with some adaptations described in [43]. The values are presented as the mean of three independent tests and the standard deviation associated to the measurements. The percentage of inhibition was calculated according to Equation (1) for D and for the standard rutin.

### 3.8. Determination of Enzymatic Activity

#### 3.8.1. Acetylcholinesterase Inhibitory Activity

AChE inhibition was performed in triplicate as described by Falé et al. [55]. Succinctly, to calculate the enzyme activity without inhibitor (100% activity), 325 µL of 50 mM Tris buffer pH 8, 100 µL of Milli-Q water and 25 µL of AChE solution (1.33 U/mL) were mixed in a cuvette and incubated, at 25 °C, for 15 min. Next 75 µL of AChI (0.33 mg/mL), 475 µL of DTNB (1.2 mg/mL of 50 mM Tris buffer pH 8 containing 0.1 M NaCl and 0.02 M MgCl_2_) were added and the absorbance was read on the spectrophotometer Schimadzu UV-160A, Japan, at 405 nm, for 4 min with 10 second intervals and the initial velocity was calculated. The same procedure was carried out with the different samples, but instead of adding 100 μL of Milli-Q water, the same volume of a solution of the various samples, at 5 mg/mL, was added. In this assay, the commercial standard used was galantamine.

#### 3.8.2. HMGR Inhibitory Activity

To determine the inhibition of the enzymatic activity of HMGR, the oxidation of nicotinamide adenine dinucleotide phosphate hydrate (NADPH) was evaluated in triplicate according to the method described by Mozzicafreddo et al. [58] with minor modifications. First, in order to calculate maximum enzyme activity (100%), the kit indications were followed. Then, aliquots were removed at 0, 1, 2, 4 and 6 min, the reaction was stopped by adding MeOH to a final volume of 50% and the samples were analyzed by HPLC–DAD using VWR-Hitachi Elite LaChrom®, referred in 4.4. The decrease in the absorbance of the NADPH was monitored at 340 nm, evaluating the decrease of the peak area over time, which allowed to make a linear regression and to obtain the reaction velocity.

### 3.9. Computational Methods

The AChE and HMGR structures (PDB IDs: AChE–1B41; HMG–3CCT) were processed as previously described [45]. The studied ligands were: gentiopicroside and a series of xanthones derivatives with combinations of hydroxyl and methoxy groups. The geometry optimizations were performed with Gaussian 09 [59] using B3LYP theory level and the 6-31G(d,p) basis set. The optimized conformations were used in the docking calculations with AutoDock suite version 4.2.6 [60]. In these calculations, AChE was treated as a rigid body with four partially flexible residues (Tyr124, Trp286, Tyr337 and Tyr341) in the enzyme active site. The flexibility of the selected residues between the C_α_ and C_γ_ carbons (two torsions per residue), as well as all possible torsions in the ligands, was set with AutoDock Tools version 1.5.6 [60,61]. 

The search space of possible interactions between ligand and receptor within the previously described [45] interaction region was explored by the AutoDock Lamarckian Genetic (LGA) algorithm. Electrostatic interactions were computed using the dielectric function of Mehler and Solmajer while hydrogen bonding terms and Van der Waals were computed using AutoDock parameters [62]. 

The 1250 LGA runs were performed using standard AutoDock parameters except for the number of individuals in the population (100) and the maximum number of energy evaluations (8 × 10^6^). In the clustering procedure a root mean squared tolerance of 1.0–1.5 Å was used on the heavy atoms of the flexible region. The representative binding modes selected were the lowest energy conformations belonging to the most populated clusters.

### 3.10. Statistical Analysis 

The results are expressed as mean ± standard deviation. The statistical differences between groups, using a probability level of *p* ≤ 0.05, were assessed by one-way variance analysis (ANOVA), available in Microsoft Office 2013^®^ software. Correlations were calculated using a regression line between the antioxidant activity measured with DPPH test and inhibitory activity of acetylcholinesterase with the total phenolic content in the fractions collected.

## 4. Conclusions

*C. erythraea* when prepared as a decoction has the ability to inhibit both AChE and HMGR activity. It also has antioxidant activity due to the presence of xanthones molecules. The docking studies helped us to understand at the molecular level and attribute which activity are due to which type of compound in the aqueous extraction. A large number of xanthones are good AChE inhibitors while secoiridoids like gentiopicroside can inhibit HMGR. This top-down approach helped us to understand how such a mixture of compounds present in the decoction can act in different fronts to produce ethno-pharmacological effects as described by people that consume this medicinal beverage. 

## Figures and Tables

**Table 1:** Identification proposal of the compounds present in the complete decoction by LC–MS/MS in ESI negative and positive mode. Peaks with a minus/plus superscript were analyzed in negative/positive mode. Compounds are indicated by retention time (Rt) order and numbered according to the appearance in each chromatogram, negative [M − H]^−^ or positive mode [M + H]^+^.

Peak	Rt (min)	[M − H]^−^/[M + H]^+^	Formula	Error (∆ ppm)	Main MS^2^ Fragment ions [*m/z*, *Attribution*, (Intensity %)]	Proposed Compound
**1** ^−^	6.9	389.1102	C_16_H_22_O_11_	−3.1	345.1221 [C_15_H_22_O_9_]^−^ (9); 209.0458 [C_10_H_10_O_5_]^−^ (9); 183.0659 [C_9_H_12_O_4_]^−^ (37); 165.0554 [C_9_H_10_O_3_]^−^ (74); 139.0763 [C_7_H_8_O_3_]^−^ (28); 121.0662 [C_8_H_10_O_1_]^−^ (100)	Secologanoside
**3** ^−^	7.7	551.1417	C_25_H_28_O_14_	−2.0	507.1517 [C_24_H_28_O_12_]^−^ (66); 393.1187 [C_19_H_22_O_9_]^−^ (80); 389.1081 [C_16_H_22_O_11_]^−^ (51); 345.1195 [C_15_H_22_O_9_]^−^ (44); 323.0793 [C_15_H_16_O_8_]^−^ (51); 301.0352 [C_14_H_22_O_7_]^−^ (40); 281.0676 [C_13_H_14_O_7_]^−^ (68); 161.0236 [C_9_H_6_O_3_]^−^ (100)	Caffeoyl-6′-secologa-noside
**4** ^−^	8.1	641.1738	C_28_H_34_O_17_	−2.3	347.0776 [C_17_H_16_O_8_]^−^ (59); 332.0543 [C_16_H_13_O_8_]^−^ (100); 301.0367 [C_15_H_11_O_7_]^−^ (23)	Di-hydroxy-tetrame-toxy -*O*-pentosyl-hexosylxanthone
**5** ^−^	8.2	301.0362	C_15_H_10_O_7_	−2.7	--	Quercetin
**6** ^−^	9.2	273.0413	C_14_H_10_O_6_	−3.1	258.0177 [C_13_H_7_O_6_]^−^ (100); 257.0102 [C_13_H_6_O_6_]^−^ (20); 230.0222 [C_12_H_7_O_5_]^−^ (10); 229.0144 [C_12_H_6_O_5_]^−^ (17)	Tri-hydroxy-monome-toxyxanthone
**7** ^−^	10.3	287.0568	C_15_H_12_O_6_	−2.5	272.0335 [C_14_H_9_O_6_]^−^ (22); 257.0096 [C_13_H_6_O_6_]^−^ (100); 229.0143 [C_12_H_6_O_5_]^−^ (22)	Di-hydroxy-dimetho-xyxanthone
**1** ^+^	7.1	375.1275	C_16_H_22_O_10_	2.9	195.0645 [C_10_H_10_O_4_]^+^ (9); 177.0539 [C_10_H_8_O_3_]^+^ (100); 167.0696 [C_9_H_10_O_3_]^+^ (17)	Swertiamarin
**2** ^+^	7.1	357.1171	C_16_H_20_O_9_	2.6	177.0538 [C_10_H_8_O_3_]^+^ (100); 163.0378 [C_9_H_6_O_3_]^+^ (70); 149.0601 [C_9_H_8_O_2_]^+^ (45)	Gentiopicroside
**3** ^+^	7.2	359.1327	C_16_H_22_O_9_	2.8	197.0798 [C_10_H_12_O_4_]^+^ (100); 179.0693 [C_10_H_10_O_3_]^+^ (50); 177.0536 [C_10_H_8_O_3_]^+^ (63); 149.0597 [C_9_H_8_O_2_]^+^ (41); 127.0420 [C_6_H_6_O_3_]^+^ (84)	Sweroside
**4** ^+^	8.1	349.0909	C_17_H_16_O_8_	2.6	334.0678 [C_16_H_13_O_8_]^+^ (7); 319.0443 [C_15_H_10_O_8_]^+^ (100); 301.0338 [C_15_H_8_O_7_]^+^ (12); 291.0496 [C_14_H_10_O_7_]^+^ (9)	Di-hydroxy-tetrame-thoxyxanthone
**6** ^+^	9.0	303.0857	C_16_H_14_O_6_	1.9	288.0621 [C_15_H_11_O_6_]^+^ (100); 273.0389 [C_14_H_8_O_6_]^+^ (19); 245.0438 [C_13_H_8_O_5_]^+^ (37)	Monohydroxy-trime-toxyxanthone
**7** ^+^	9.1	305.0643	C_15_H_12_O_7_	4.2	290.0416 [C_14_H_9_O_7_]^+^ (86) 275.0180 [C_13_H_6_O_7_]^+^ (100) 247.0238 [C_12_H_6_O_6_]^+^ (30)	Tri-hydroxy-dimeto-xyxanthone

**Table 2:** Total phenols and bioactivities of the complete decoction (D) of *C. erythraea*, the decoction mucilage free (DM_f_), the xanthones fraction (XF) and the standard gentiopicroside.

Extracts/Standard	Total Phenols (μg GAE/mg of Extract)	Antioxidant Activity			Enzyme Inhibitory Activity	
		DPPH (50 μg/mL) (%)	TBARS (400 μg/mL) (%)	EC_50_ NO’s (μg/mL)	AChE (500 μg/mL) (%)	HMGR (10 μg/mL) (%)
D	22.37 ± 0.36	25.25 ± 0.72	32.23 ± 0.93	774.9 ± 13.8	56.43 ± 0.84	47.99 ± 0.47
DM_f_	18.82 ± 0.25	21.95 ± 0.46	-	-	47.09 ± 0.95	-
XF	16.31 ± 0.25	17.63 ± 0.41	-	-	59.81 ± 0.7	26.86 ± 0.87
Gentio-picroside	-	1.31±0.53	-	-	6.51 ± 0.18	57.95 ± 0.51

**Table 3:** Summary of all xanthones used in the AChE molecular docking calculations with their binding energies and inhibition constants. The R1-R8 positions relate to the xanthone template (Figure 3) and the OH/OMe labels refer to the hydroxyl and methoxy groups. The peaks refer to the positive and negative ESI modes in Table 1.

Final Name	Peak	R_1_	R_2_	R_3_	R_4_	R_5_	R_6_	R_7_	R_8_	ΔG_BIND_ (kcal/mol)	K_i_ (μM)
xan01 *	4^+^	OH		OMe		OMe	OMe	OMe	OH	−6.0	36.9
xan02 *	4^+^	OH		OMe		OMe	OH	OMe	OMe	−5.6	77.6
xan03 *	6^+^	OH	OMe	OMe		OMe				−6.9	8.9
xan04 *	6^+^	OMe		OH		OMe	OMe			−6.6	15.3
xan05 * (decussatin)	6^+^	OH		OMe				OMe	OMe	−5.7	65.6
xan06 ^*^	6^+^	OH		OMe		OMe	OMe			−5.9	50.9
xan07 *	7^+^	OH	OMe	OH		OMe	OH			−6.1	35.1
xan08 *	6^−^	OH		OMe		OH	OH			−7.5	2.9
xan09 * (bellidifolin)	6^−^	OH		OMe		OH			OH	−7.5	2.9
xan10 *	6^−^	OH	OMe	OH		OH				−6.5	17.0
xan11 *	7^−^	OH		OMe		OMe	OH			−7.1	5.9
xan12	4^+^	OH	OMe		OMe	OMe		OMe	OH	−7.5	3.4
xan13	7^+^		OH		OH	OH		OMe	OMe	−6.4	19.8
xan14	6^−^	OH				OH	OMe	OH		−5.9	50.0
xan15	6^−^	OH			OH	OH		OMe		−7.5	3.0
xan08.1		OH		OH		OH	OH			−7.3	4.2
xan08.2		OH				OH	OH			−7.4	3.9
xan08.3		OH		OMe	OH	OH	OH			−7.1	6.0
xan09.1		OH		OH		OH			OH	−7.4	3.5
xan09.2		OH				OH			OH	−6.8	10.1
xan12.1		OH			OMe	OMe		OMe	OH	−7.9	1.6
xan12.2		OH	OH		OMe	OMe		OMe	OH	−7.2	5.2
xan12.3		OH	OMe		OH	OMe		OMe	OH	−7.4	4.0
xan12.4		OH	OMe		OMe	OMe		OH	OH	−7.3	4.4
xan15.1	7^−^	OMe			OH	OH		OMe		−6.7	12.3

* The position of substituent groups on the xanthone structure (Figure 2) indicated in this table was indicated in the literature: [15] for xan01, 02, 03, 04, 07, 08, 09, 10 and 11; [46] for xan01, 04, 05, 06 and 10; [47] for xan11.

**Table 4:** Summary of all compounds used in the HMGR molecular docking calculations with their binding energies (ΔG_BIND_) and inhibition constants (K_i_).

Name	ΔG_BIND_ (kcal/mol)	K_i_ (μM)
xan01	−5.7	70.6
xan03	−5.5	98.9
xan04 (bellidifolin)	−5.3	129.6
xan07	−5.1	184.8
xan13	−5.6	78.1
xan08	−5.6	78.1
xan05	−5.5	94.1
xan12	−5.7	71.8
xan10	−5.2	150.9
xan14	−5.7	70.6
xan15	−5.3	129.6
xan06	−5.3	131.8
xan02	−5.8	54.8
xan11	−5.4	102.3
xan09	−5.6	74.3
gentiopiocroside	−6.4	19.9
statin	−11.5	3.5 × 10^−3 a)^

^a)^ This is a corrected K_i_ value and is different from the one reported in [52], which had an error in the formula used.
